# Supplementary material for: Structural basis for late maturation steps of the human mitoribosomal large subunit
Source: Nat Commun. 2021 Jun 16;12:3673. doi: 10.1038/s41467-021-23617-8 (PMC8209036; doi:10.1038/s41467-021-23617-8)
Supplement: Supplementary file 1 — Supplementary Information [file 41467_2021_23617_MOESM1_ESM.pdf]

SUPPLEMENTARY INFORMATION

Structural basis for late maturation steps of the human mitoribosomal large subunit

Cipullo, Valentín Gesé et al.

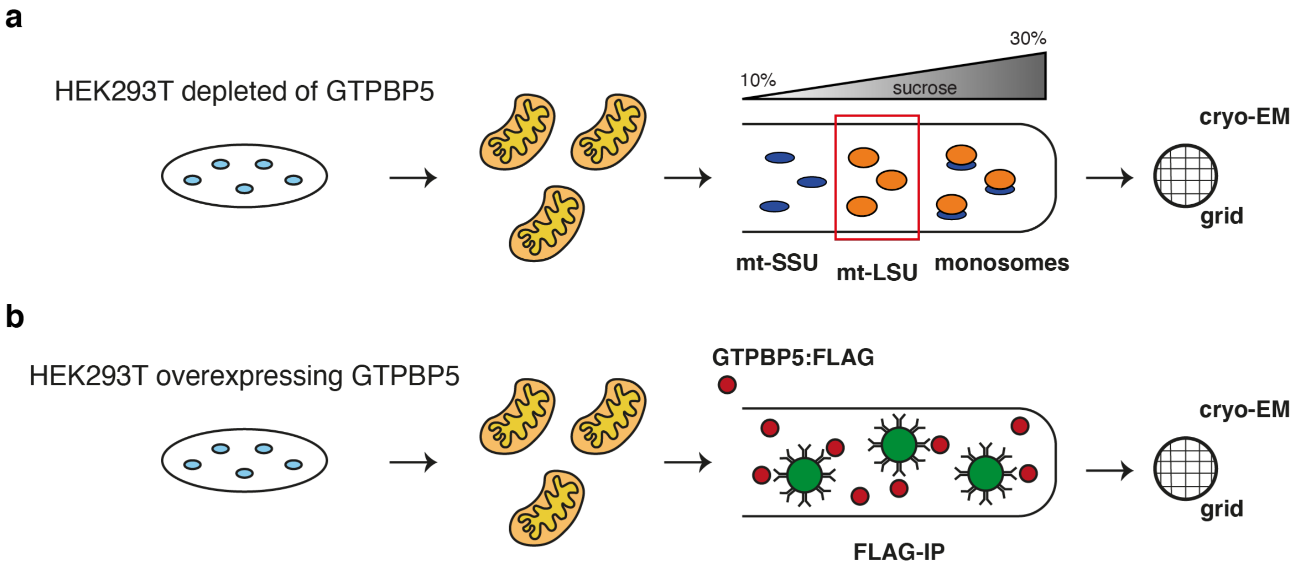

Extended Data Fig. 1 Experimental approaches employed to purify mitoribosome assembly intermediates.
a Sucrose gradient centrifugation experiment was used to determine GTPBP5^{KO} mt-LSU intermediate structure. **b** FLAG-immunoprecipitation experiment was performed to determine GTPBP5^{IP} mt-LSU intermediate structure.

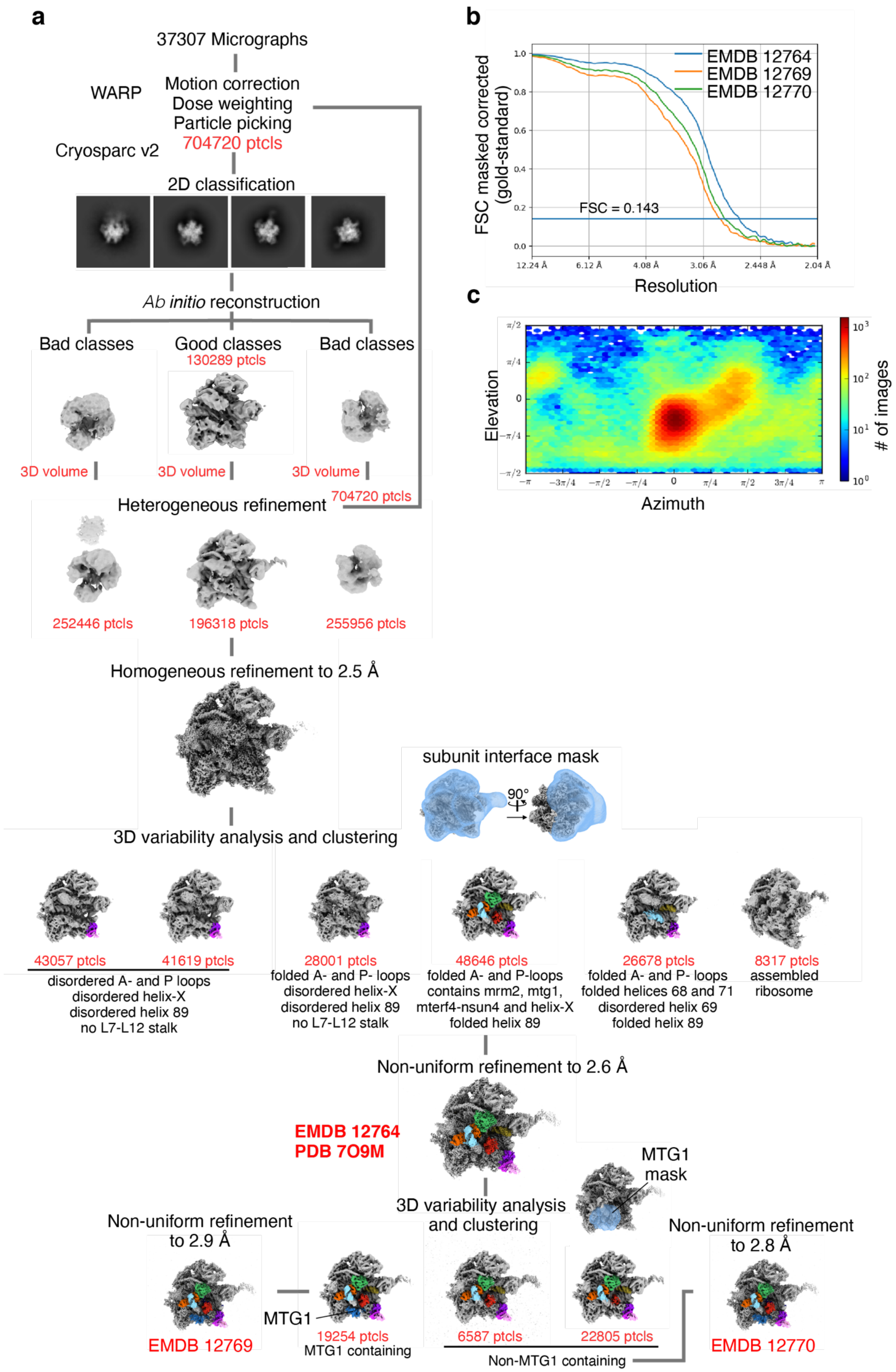
a
37307 Micrographs
WARP
Motion correction
Dose weighting
Particle picking
704720 ptcls
Cryosparc v2
2D classification
Ab initio reconstruction
Bad classes
Good classes
130289 ptcls
Bad classes
3D volume
3D volume
3D volume
Heterogeneous refinement
704720 ptcls
252446 ptcls
196318 ptcls
255956 ptcls
Homogeneous refinement to 2.5 Å
subunit interface mask
90°
3D variability analysis and clustering
43057 ptcls
41619 ptcls
disordered A- and P loops
disordered helix-X
disordered helix 89
no L7-L12 stalk
28001 ptcls
folded A- and P- loops
disordered helix-X
disordered helix 89
no L7-L12 stalk
48646 ptcls
folded A- and P-loops
contains mrm2, mtg1,
mterf4-nsun4 and helix-X
folded helix 89
26678 ptcls
folded A- and P- loops
folded helices 68 and 71
disordered helix 69
folded helix 89
8317 ptcls
assembled
ribosome
Non-uniform refinement to 2.6 Å
EMDB 12764
PDB 7O9M
MTG1
mask
3D variability analysis
and clustering
Non-uniform refinement
to 2.9 Å
EMDB 12769
MTG1
19254 ptcls
MTG1 containing
6587 ptcls
22805 ptcls
Non-MTG1 containing
Non-uniform refinement
to 2.8 Å
EMDB 12770
b
FSC masked corrected
(gold-standard)
EMDB 12764
EMDB 12769
EMDB 12770
FSC = 0.143
1.0
0.8
0.6
0.4
0.2
0.0
12.24 Å
6.12 Å
4.08 Å
3.06 Å
2.448 Å
2.04 Å
Resolution
c
Elevation
π/2
π/4
0
−π/4
−π/2
−π
−3π/4
−π/2
−π/4
0
π/4
π/2
3π/4
π
Azimuth
10^3
10^2
10^1
10^0
# of images

Extended Data Fig. 2 Data processing strategy for the GTPBP5^{KO} dataset.
a Data processing strategy for the three deposited reconstructions (EMDB 12764, 12769 and 12770) from the GTPBP5^{KO} dataset. Colouring as in Figure 1. For the 3D variability analysis, the mask used is shown as a blue semi-transparent surface. **b** Gold-standard Fourier shell correlation (FSC) [1] for the three EMDB-deposited reconstructions. The horizontal blue line indicates the FSC cut-off at 0.143. **c** Heatmap of the angular distribution for particle projections after heterogeneous refinement (196318 particles).

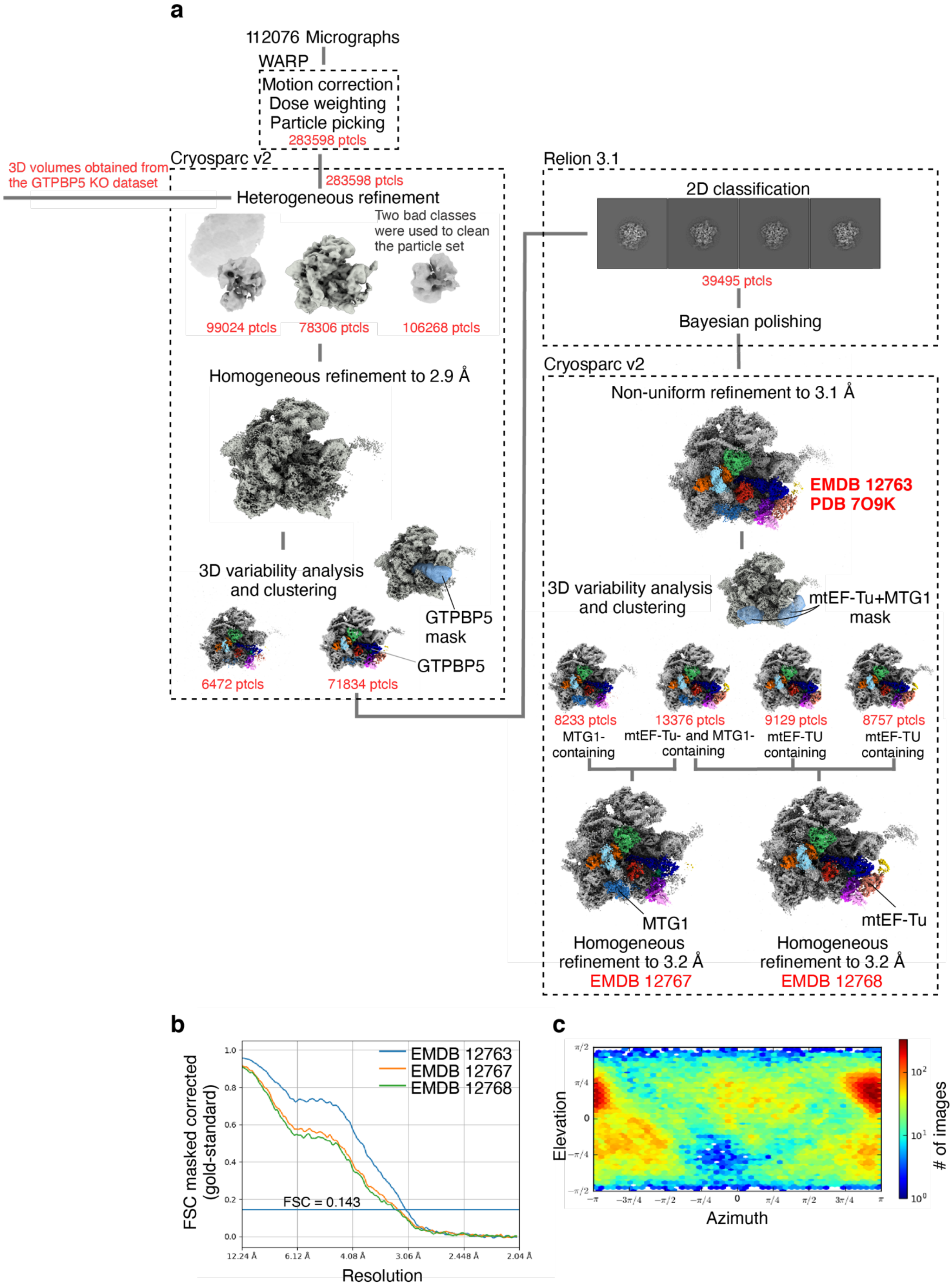

Extended Data Fig. 3 Data processing strategy for the GTPBP5^{IP} dataset.
a Data processing strategy for the three deposited reconstructions (EMDB 12763, 12767 and 12768) from the GTPBP5^{IP} dataset. Colouring as in Figure 1. For the 3D variability analysis, the mask used is shown as a blue semi-transparent surface. **b** Gold-standard Fourier shell correlation (FSC) [1] for the

three EMDB-deposited reconstructions. The horizontal blue line indicates the FSC cut-off at 0.143. **c** Heatmap of the angular distribution for particle projections after homogeneous refinement (78306 particles).

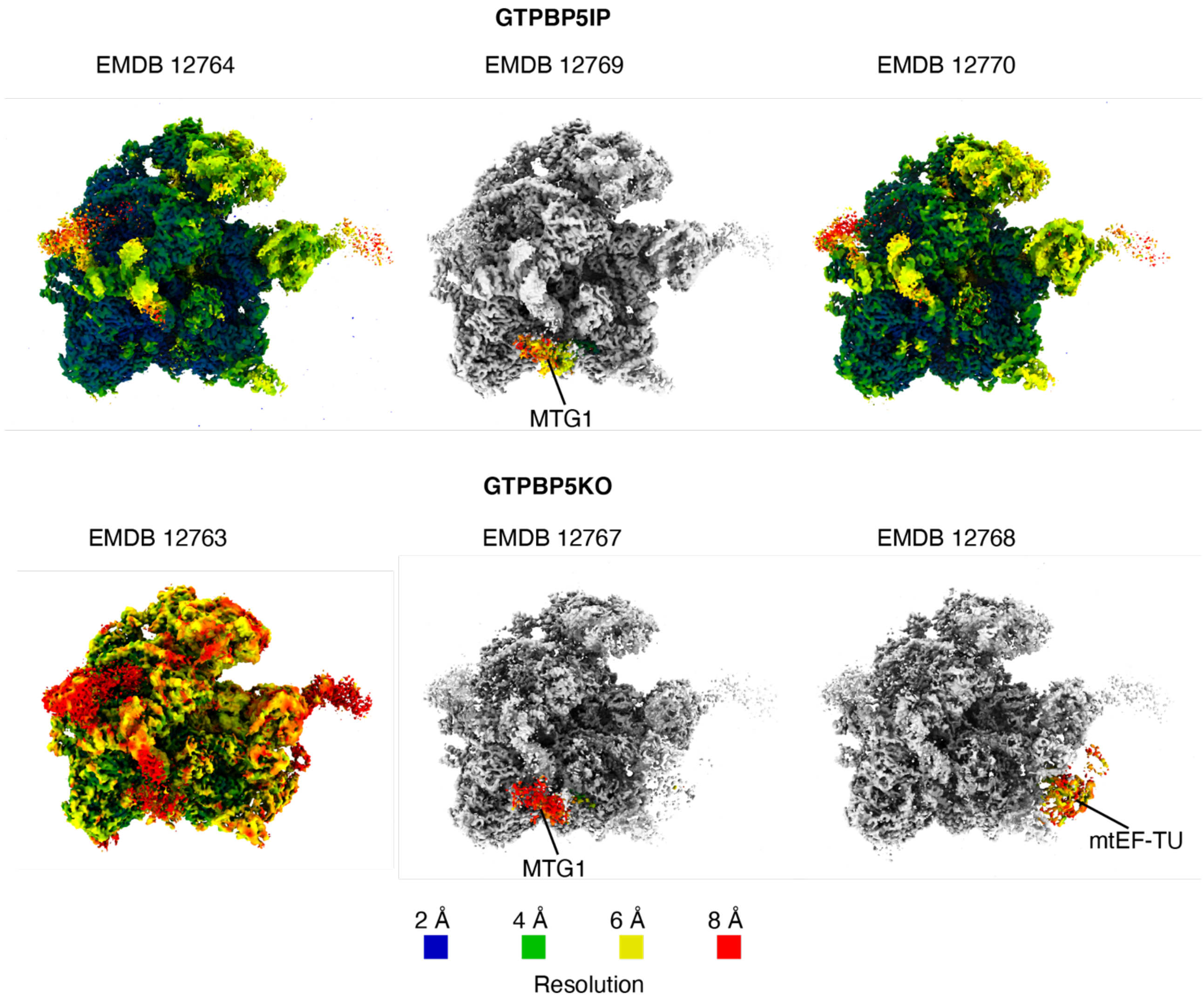

Extended Data Fig. 4.
Local resolution of the deposited reconstructions indicated by colouring from blue (2 Å) to red (8 Å). The local resolution was estimated in CryoSPARC. For the reconstructions derived from the MTG1 or mtEF-Tu containing subsets, the non-relevant parts have been grayed out.

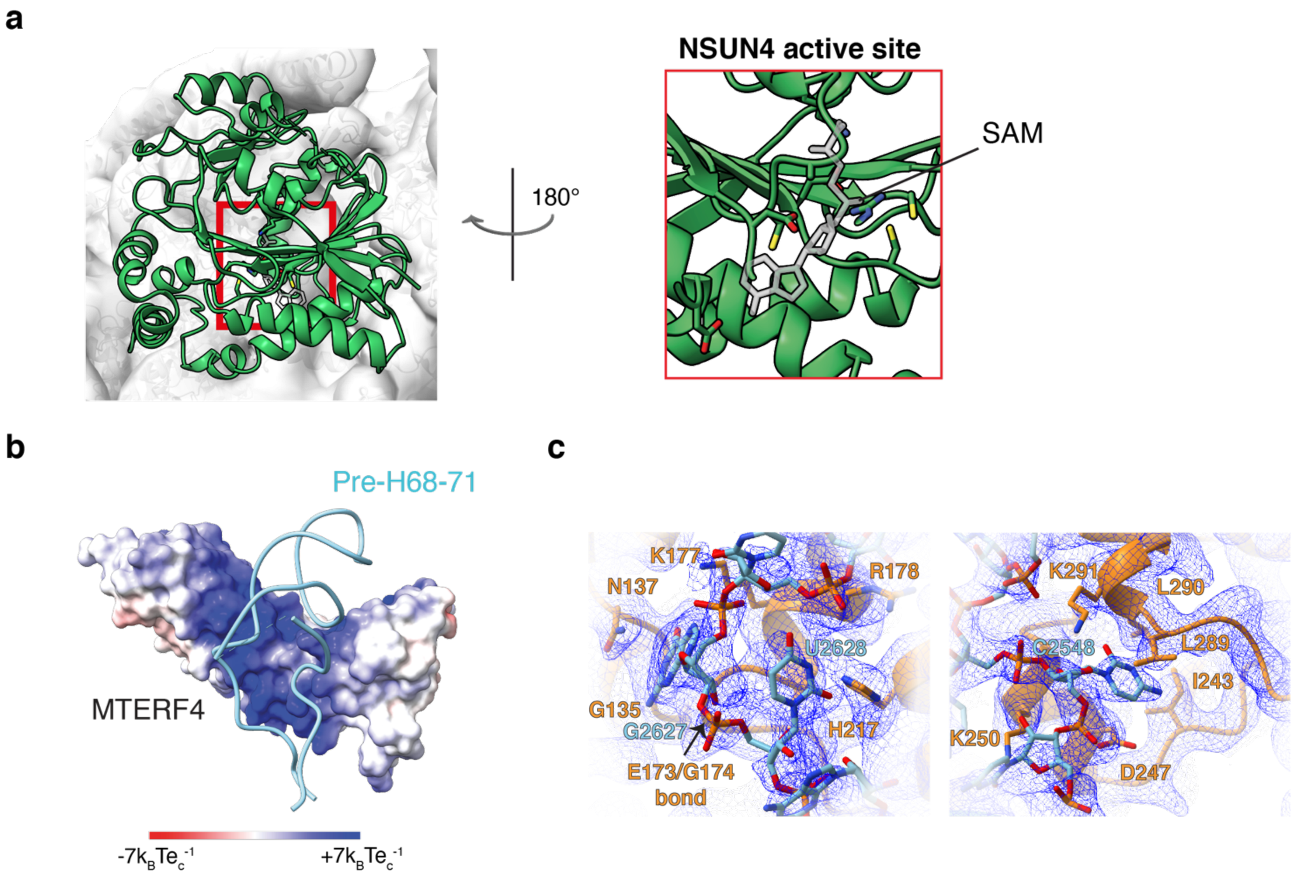

Extended Data Fig. 5 NSUN4 active site and MTERF4-pre-H68-71 interactions.

a View of NSUN4 in both GTPBP5^{KO} and GTPBP5^{IP} structures facing the mt-LSU. NSUN4 active site is indicated by a red rectangle. The right zoomed-in panel obtained after 180°C rotation in respect to the left panel shows the residues involved in SAM coordination and SAM as sticks. **b** View of the electrostatic potential on MTERF4 surface interacting with pre-H68-71. Red and blue refer to electronegative and electropositive regions, respectively. ABPS was used to calculate the electrostatic potential [2]. **c** Quality of the map density for relevant interactions between MTERF4 and pre-H68-71 (related to figure 2b). Contour level at 0.13.

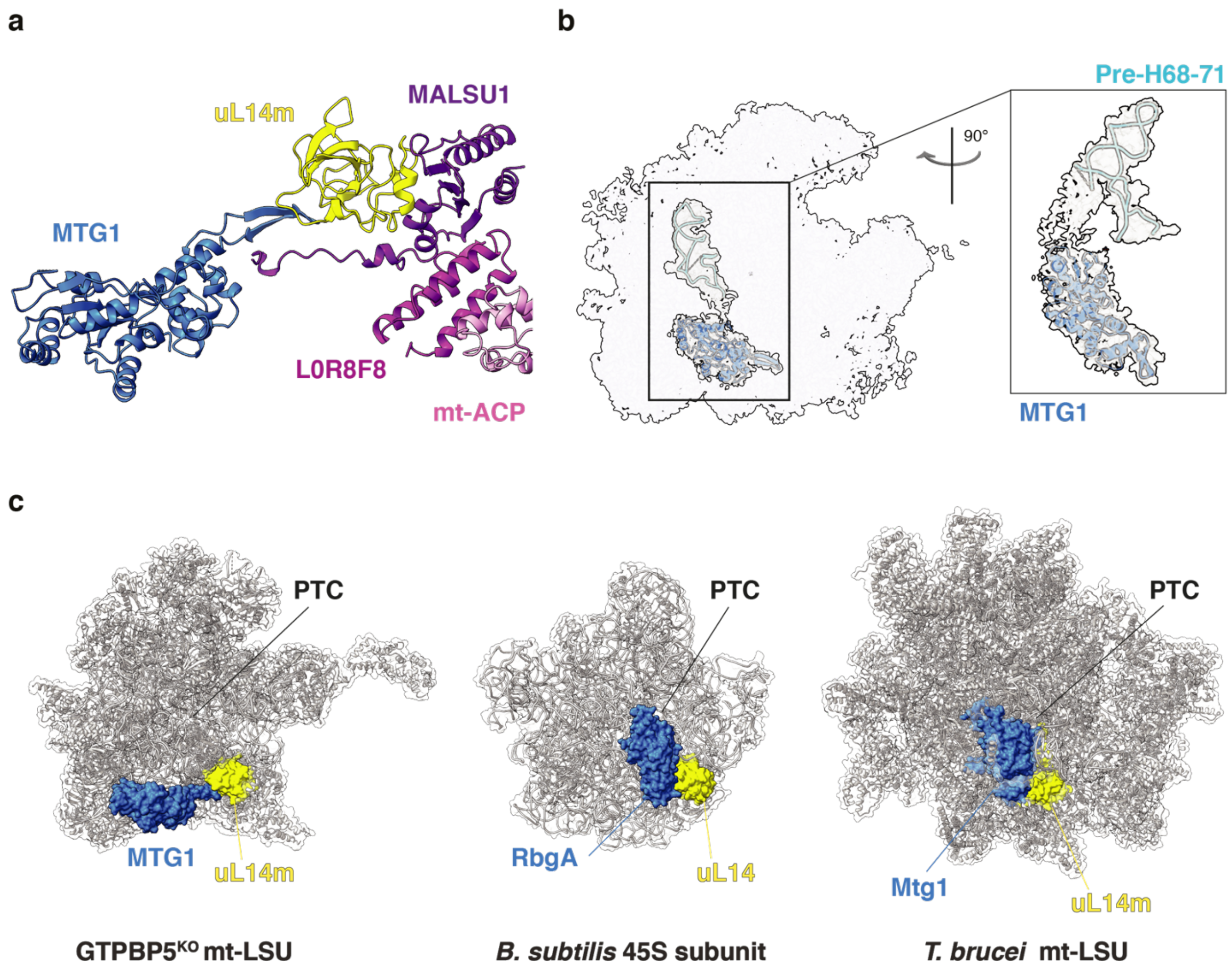

Extended Data Fig. 6 Structural features of MTG1 and the MALSU1 module.
a Cartoon representation of MALSU1 contacting MTG1. L0R8F8, mt-ACP and uL14m are also shown. **b** Cryo-EM density of the MTG1 and pre-H68-71 contact on the GTPBP5$^{KO/IP}$ mt-LSU. The rotated zoomed-in panel shows the density of MTG1 contacted by the bottom part of pre-H68-71 that was not modelled due to the lower resolution. Pre-H68-71 is represented in light blue while MTG1 is represented in pale blue. **c** Comparison of MTG1 position on the GTPBP5$^{KO/IP}$ mt-LSU, with its homologues RbgA on the *B. subtilis* 45S subunit (PDB: 6PPK [3]) and Mtg1 on the *T. brucei* mt-LSU (PDB: 6YXY [4]). Mitoribosomal protein uL14m that is situated in close proximity to MTG1 is coloured in yellow, while the PTC region is indicated with a label. MTG1, RbgA and Mtg1 are indicated in pale blue colour.

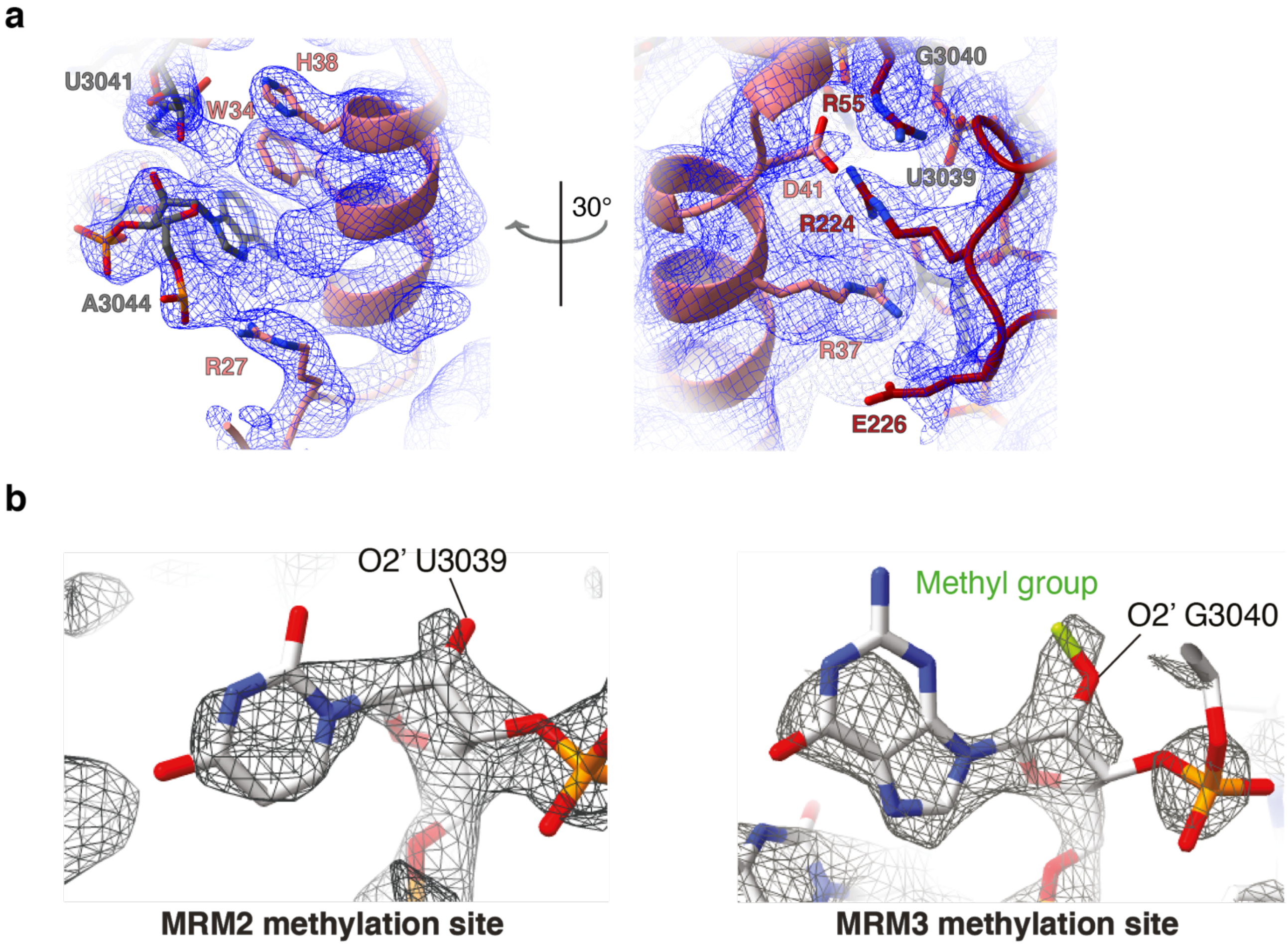

Extended Data Fig. 7 MRM2-A-loop interaction network and MRM2 and MRM3 methylation sites on the mt-LSU.
a Quality of the map density for the interactions involving the MRM2 N-terminal helix (related to figure 2e). Contour level at 0.14. **b** Representation of MRM2 (U3039; left) and MRM3 (G3040; right) methylation sites. The 2′-O of the ribose is highlighted in both views. The methyl group of MRM3 methylation site is indicated in green (right). The methyl group is missing in MRM2 methylation site (left). For the density representation, the map was sharpened with a B-factor of -20 in CryoSPARC. Contour level at 0.581.

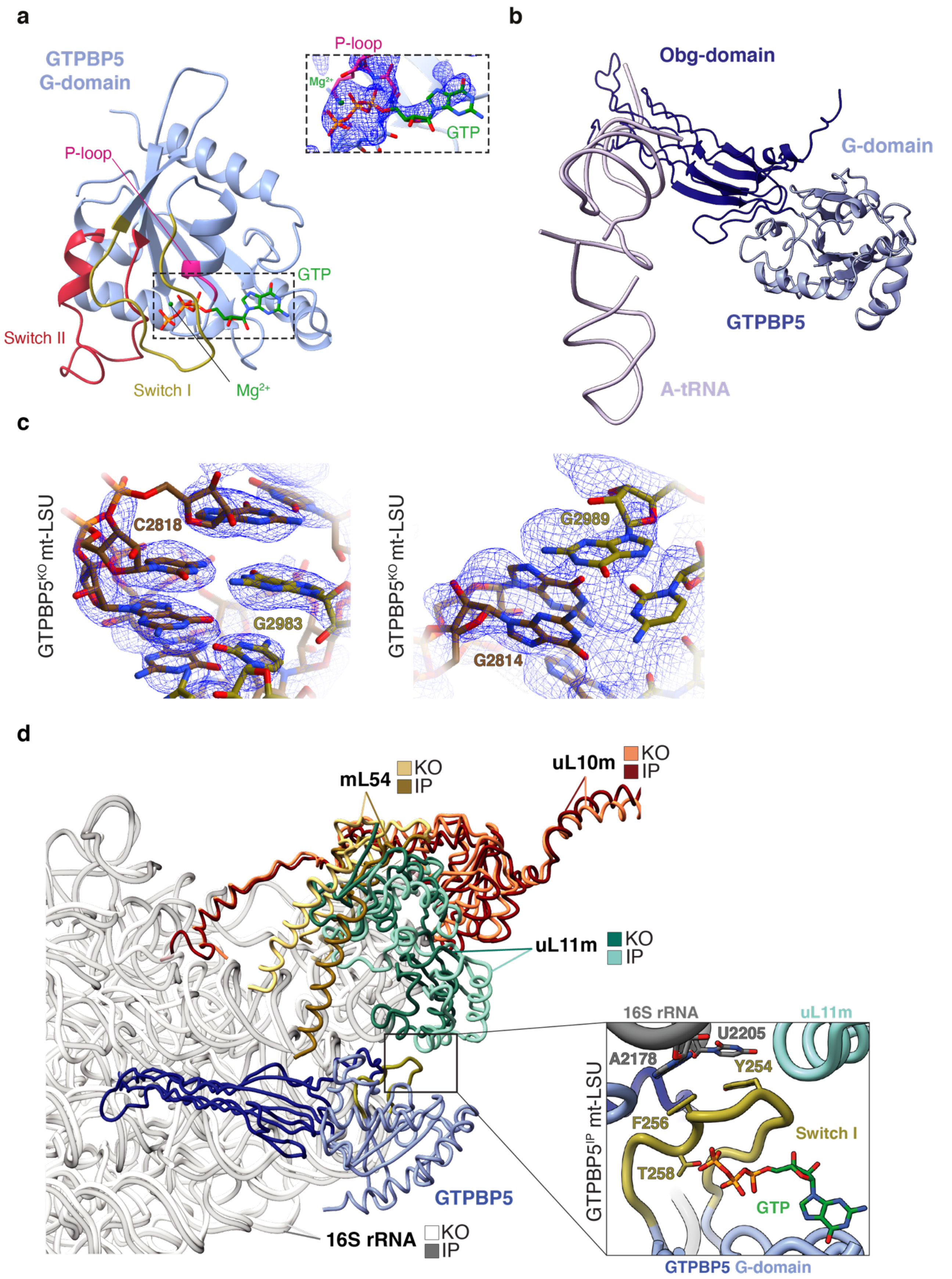

Extended Data Fig. 8 Structural features of GTPBP5 and its interaction with the mt-LSU, and structural details of H89-P-loop interaction in GTPBP5^{KO} structure.

a GTPBP5 G-domain displays GTP (green) in its binding pocket. Switch I, Switch II, the P-loop and the Mg^{2+} are indicated. In the right-upper panel, GTP corresponding density map is indicated in blue.

Contour level at 0.22. **b** Comparison of the GTPBP5^{IP} structure with the translating mitoribosome (PDB: 5AJ4 [5]) shows that GTPBP5 overlaps with the A-site tRNA. **c** Quality of the map density for the interactions between the P-loop and H89 in GTPBP5^{KO} mt-LSU structure (related to Fig. 3d). Contour level at 0.25 for the left panel and at 0.17 for the right panel. **d** Representation of the L7/L12 stalk movement in GTPBP5^{IP} mt-LSU (IP) when compared to the GTPBP5^{KO} mt-LSU (KO). The 16S mt-rRNA, mL54, uL10m and uL11m are highlighted in both structures. GTPBP5 is also shown. The right zoomed-in panel features interactions between GTPBP5 G-domain and the 16S mt-rRNA in GTPBP5^{IP} mt-LSU structure. The G-domain Switch I and GTP are shown in yellow and green, respectively.

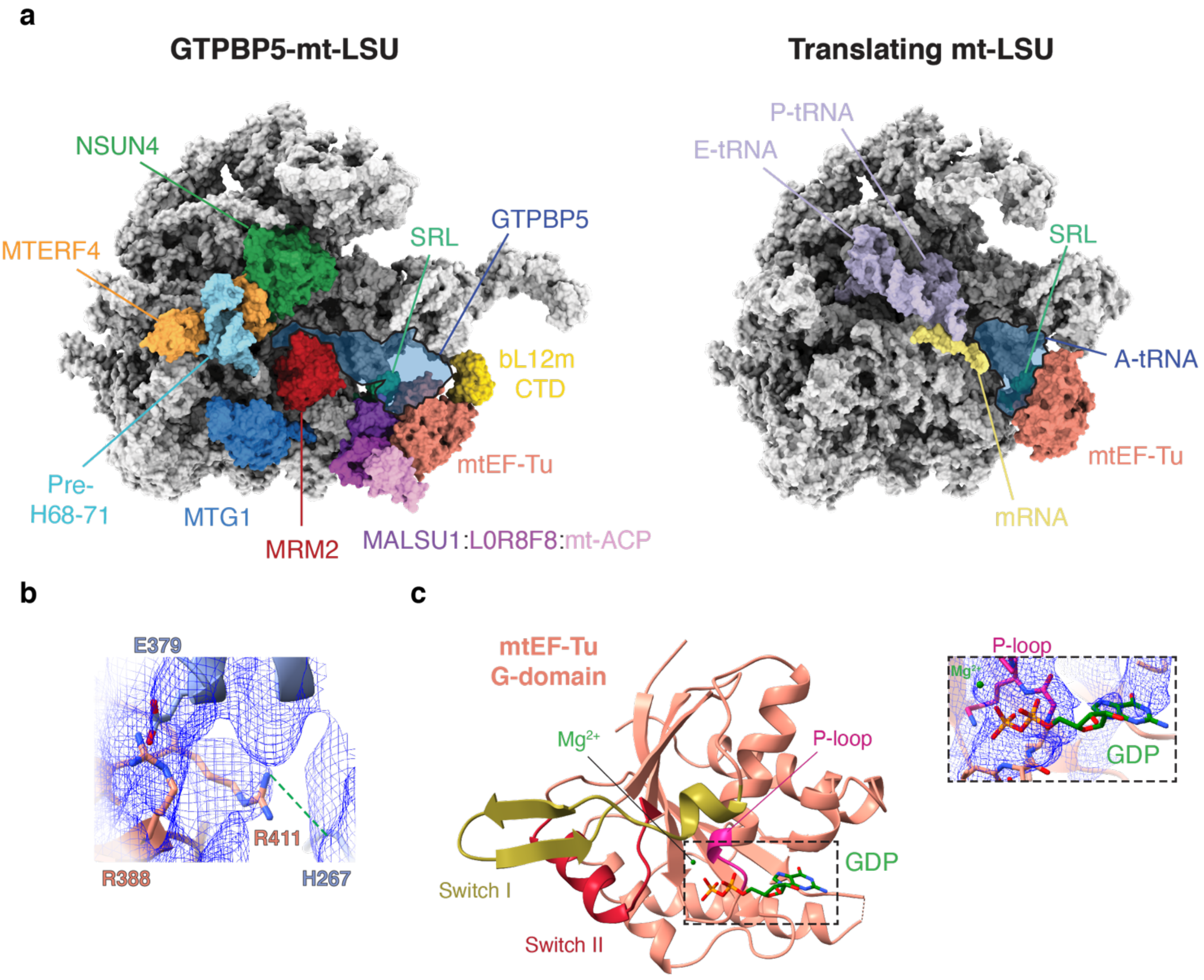

Extended Data Fig. 9 Structural features of mtEF-Tu interaction with the mt-LSU during mitoribosome assembly and translation.
a Surface representation of the GTPBP5^{IP} mt-LSU and the translating mt-LSU (PDB: 7A5G [6]) showing the different position of mtEF-Tu. In GTPBP5^{IP} mt-LSU structure, mtEF-Tu, MTERF4-NSUN4, MRM2, MTG1, GTPBP5, MALSU1 module, bL12 CTD, pre-H68-71 and SRL are indicated. In the translating mt-LSU structure the A-, P- and E-tRNAs are shown together with mtEF-Tu, SRL and mRNA (PDB: 7A5G [6]). GTPBP5 and the A-site tRNA in the respective structures are shown semi-trasparent. **b** Quality of the map density for the interactions between mtEF-Tu and GTPBP5 in GTPBP5^{IP} mt-LSU structure (related to Fig. 4c). Contour level at 0.13. **c** mtEF-Tu G-domain displaying GDP (green) in its binding pocket. Switch I, Switch II, the P-loop and the Mg^{2+} are indicated. In the right-upper panel, GDP corresponding density map is indicated in blue. Contour level at 0.15.

Extended Data Table 1 Cryo-EM data collection, refinement and validation statistics

	GTPBP5^{IP}	GTPBP5^{KO}
	(EMDB-12763)<br>(EMDB-12767 for class with 100% MTG1 occupancy)<br>(EMDB-12768 for class with 100% mtEF-TU occupancy)<br>(PDB-7O9K)	(EMDB-12764)<br>(EMDB-12769 for class with 100% MTG1 occupancy)<br>(EMDB-12770 for class with no MTG1)<br>(PDB-7O9M)
**Data collection and processing**		
Microscope	FEI Titan Krios G3i	
Detector	K3	
Voltage (kV)	300	
Electron exposure (e^{-}/Å^2/sec)	49	
Energy filter slit width (eV)	10	
Pixel size (Å)	0.51	
SA EFTEM Mag.	165,000	
Defocus range (µm)	-0.3 to -1.1	
Map Resolution at FSC = 0.143 (Å)	3.1 for EMDB-12763<br>3.2 for EMDB-12767<br>3.2 for EMDB-12768	2.6 for EMDB-12764<br>2.9 for EMDB-12769<br>2.9 for EMDB-12770
Sharpening B factor (Å^2)	-35.47 for EMDB-12763<br>-12.2 for EMDB-12767<br>-10.1 for EMDB-12768	-36.7 for EMDB-12764<br>-25.5 for EMDB-12769<br>-31.0 for EMDB-12770
**Refinement**		
Initial model used (PDB code)	5OOL	5OOL
Model resolution at FSC = 0.143 (Å)	3.1	2.6
Model Composition		
No. of chains	76	71
Total atoms	115687	110356
Protein residues	10324	9573
RNA residues	1498	1515
Ligands: GTP/GDP/SAM/SAH/PNS/Mg^{2+}/Zn^{2+}	1/2/1/1/1/93/2	0/1/1/0/1/112/2
B Factors (min/max/mean) (Å^2)		
Protein	40.0/640.0/190.9	14.2/628.0/102.6
RNA	40.0/640.0/165.4	4.12/625.4/74.8
Ligands	38.2/420.6/250.6	19.1/317.7/183.8
Validation		
RMSD bonds (ouliers) (Å)	0.009 (9)	0.009 (2)
RMSD angles (outliers) (°)	0.898 (11)	0.910(19)
Clashscore	10.68	8.58
MolProbity score	1.65	1.52
Rotamer outliers (%)	0.071	0.46
Ramachandran plot		
Favored/Allowed/Outliers (%)	97.29/2.69/0.02	97.73/2.23/0.03
RNA Validation		
Sugar pucker outliers (%)	1.67	1.65
Angle/bond outliers (%)	0.1	0
Bond outliers (%)	0	0

EMDB, Electron Microscopy Data Bank; PDB, Protein Data Bank; RMSD, root-mean-square deviation; FSC, Fourier shell correlation.GTP, guanosine triphosphate; GDP, guanosine diphosphate; SAH, S-adenosylhomocysteine; SAM, S-adenosyl methionine; PNS 4'-phosphopantetheine

Extended Data Table 2 Protein and RNA components of the mt-LSU assembly intermediates.

Chain	Modelled residues GTPBP5^{KO}	Modelled residues GTPBP5^{IP}	UniprotID	Description	short name
0	79-200	79-200	Q9BYC8	39S ribosomal protein L32, mitochondrial, L32mt, MRP-L32	RM32_HUMAN
1	14-65	14-65	O75394	39S ribosomal protein L33, mitochondrial, L33mt, MRP-L33	RM33_HUMAN
2	48-92	48-92	Q9BQ48	39S ribosomal protein L34, mitochondrial, L34mt, MRP-L34	RM34_HUMAN
3	94-188	94-188	Q9NZE8	39S ribosomal protein L35, mitochondrial, L35mt, MRP-L35	RM35_HUMAN
4	66-200	66-103	Q9P0J6	39S ribosomal protein L36, mitochondrial, L36mt, MRP-L36	RM36_HUMAN
5	31-422	31-422	Q9BZE1	39S ribosomal protein L37, mitochondrial, L37mt, MRP-L37	RM37_HUMAN
6	27-79,99-209,213-282,291-380	27-79,99-209,213-380	Q96DV4	39S ribosomal protein L38, mitochondrial, L38mt, MRP-L38	RM38_HUMAN
7	36-322	36-325	Q9NYK5	39S ribosomal protein L39, mitochondrial, L39mt, MRP-L39	RM39_HUMAN
8	97-181	97-181	Q9NQ50	39S ribosomal protein L40, mitochondrial, L40mt, MRP-L40	RM40_HUMAN
9	15-137	15-137	Q8IXM3	39S ribosomal protein L41, mitochondrial, L41mt, MRP-L41	RM41_HUMAN
A	1671-3388	1671-3386	NA	16S RRNA	16S RRNA
A1	36-108,123-384, SAM	27-108,123-384, SAM	Q96CB9	5-methylcytosine rRNA methyltransferase NSUN4	NSUN4_HUMAN
B	1603-1670	1603-1670	NA	MT-TRNAVAL	MT-TRNAVAL
A2	90-327	90-327	Q7Z6M4	Transcription termination factor 4, mitochondrial	MTEF4_HUMAN
C	55-185,213-401, GDP	55-185,213-401, GDP	Q9BT17	Mitochondrial ribosome-associated GTPase 1	MTG1_HUMAN
D	61-401	61-401	Q5T653	39S ribosomal protein L2, mitochondrial, L2mt, MRP-L2	RM02_HUMAN
t1	46-92	46-92	P52815	39S ribosomal protein L12, mitochondrial	RM12_HUMAN
E	45-348	45-348	P09001	39S ribosomal protein L3, mitochondrial, L3mt, MRP-L3	RM03_HUMAN
t2	62-91	62-91	P52815	39S ribosomal protein L12, mitochondrial	RM12_HUMAN
F	45-294	45-294	Q9BYD3	39S ribosomal protein L4, mitochondrial, L4mt, MRP-L4	RM04_HUMAN
G	Not present	59-402, GTP	Q9H4K7	Mitochondrial ribosome-associated GTPase 2 (GTP-binding protein 5)	MTG2_HUMAN
H	53-147	53-147	Q9BYD2	39S ribosomal protein L9, mitochondrial, L9mt, MRP-L9	RM09_HUMAN
I	36-240	29-240	Q7Z7H8	39S ribosomal protein L10, mitochondrial, L10mt, MRP-L10	RM10_HUMAN
J	18-157	18-157	Q9Y3B7	39S ribosomal protein L11, mitochondrial, L11mt, MRP-L11	RM11_HUMAN
K	2-178	2-178	Q9BYD1	39S ribosomal protein L13, mitochondrial, L13mt, MRP-L13	RM13_HUMAN
L	31-145	31-145	Q6P1L8	39S ribosomal protein L14, mitochondrial, L14mt, MRP-L14	RM14_HUMAN
M	10-301	10-301	Q9P015	39S ribosomal protein L15, mitochondrial, L15mt, MRP-L15	RM15_HUMAN
N	32-133,148-251	62-134,146-251	Q9NX20	39S ribosomal protein L16, mitochondrial, L16mt, MRP-L16	RM16_HUMAN
O	9-160	9-160	Q9NRX2	39S ribosomal protein L17, mitochondrial, L17mt, MRP-L17	RM17_HUMAN
P	39-179	39-179	A8K9D2	Mitochondrial ribosomal protein L18, isoform CRA_b	A8K9D2_HUMAN
Q	74-290	74-290	P49406	39S ribosomal protein L19, mitochondrial, L19mt, MRP-L19	RM19_HUMAN
R	10-149	10-149	Q9BYC9	39S ribosomal protein L20, mitochondrial, L20mt, MRP-L20	RM20_HUMAN
S	49-204	49-204	Q7Z2W9	39S ribosomal protein L21, mitochondrial, L21mt, MRP-L21	RM21_HUMAN
T	47-301	47-301	E7ESL0	39S ribosomal protein L22, mitochondrial	E7ESL0_HUMAN
U	2-112,126-153	2-112,126-153	Q16540	39S ribosomal protein L23, mitochondrial, L23mt, MRP-L23	RM23_HUMAN
V	15-216	15-216	Q96A35	39S ribosomal protein L24, mitochondrial, L24mt, MRP-L24	RM24_HUMAN
W	48-201	48-201	Q9P0M9	39S ribosomal protein L27, mitochondrial, L27mt, MRP-L27	RM27_HUMAN
X	2-244	2-244	Q13084	39S ribosomal protein L28, mitochondrial	RM28_HUMAN
Y	63-238	63-238	Q9HD33	39S ribosomal protein L47, mitochondrial, L47mt, MRP-L47	RM47_HUMAN
Z	35-154	35-154	Q8TCC3	39S ribosomal protein L30, mitochondrial, L30mt, MRP-L30	RM30_HUMAN
a	35-77,104-142	35-77,104-142	Q9Y6G3	39S ribosomal protein L42, mitochondrial, L42mt, MRP-L42	RM42_HUMAN
b	2-149	2-149	Q8N983	39S ribosomal protein L43, mitochondrial, L43mt, MRP-L43	RM43_HUMAN
c	31-316	31-107,119-316	Q9H9J2	39S ribosomal protein L44, mitochondrial, L44mt, MRP-L44	RM44_HUMAN
d	36-54,69-91,117-294	36-52,70-91,111-294	Q9BRJ2	39S ribosomal protein L45, mitochondrial, L45mt, MRP-L45	RM45_HUMAN
f	48-66,77-132,150-212	48-66,77-132,150-193,196-212	Q96GC5	39S ribosomal protein L48, mitochondrial, L48mt, MRP-L48	RM48_HUMAN
e	43-104,116-217,227-279	43-104,116-217,227-279	Q9H2W6	39S ribosomal protein L46, mitochondrial, L46mt, MRP-L46	RM46_HUMAN
g	36-201	38-201	Q13405	39S ribosomal protein L49, mitochondrial, L49mt, MRP-L49	RM49_HUMAN
h	51-78,82-158	52-158	Q8N5N7	39S ribosomal protein L50, mitochondrial, L50mt, MRP-L50	RM50_HUMAN
i	32-128	32-128	Q4U2R6	39S ribosomal protein L51, mitochondrial, L51mt, MRP-L51	RM51_HUMAN
j	24-108	24-108	A8K7J6	39S ribosomal protein L52, mitochondrial	A8K7J6_HUMAN
k	2-96	13-56,61-96	Q96EL3	39S ribosomal protein L53, mitochondrial, L53mt, MRP-L53	RM53_HUMAN
l	1-26,94-136	1-26,94-136	Q6P161	39S ribosomal protein L54, mitochondrial	RM54_HUMAN
m	34-78	34-78	Q7Z7F7	39S ribosomal protein L55, mitochondrial	RM55_HUMAN
n	25-239	51-239, SAH	Q9UI43	rRNA methyltransferase 2, mitochondrial (Homo sapiens)	MRM2_HUMAN
p	38-61,70-83,95-163,174-193	38-61,70-83,95-163,174-193	Q14197	Peptidyl-tRNA hydrolase ICT1, mitochondrial, EC 3.1.1.29	ICT1_HUMAN
o	23-102	12-102	Q9BQC6	Ribosomal protein 63, mitochondrial, hMRP63	RT63_HUMAN
q	25-159	25-159	Q8TAE8	Growth arrest and DNA damage-inducible proteins-interacting protein 1	G45IP_HUMAN
r	35-41,47-196	35-41,47-196	Q9NVS2	39S ribosomal protein S18a, mitochondrial, MRP-S18-a, Mrps18a	RT18A_HUMAN
s	41-119,138-430	41-119,140-430	Q9NP92	39S ribosomal protein S30, mitochondrial, MRP-S30, S30mt	RT30_HUMAN
t	Not present	55-184,190-440, GDP	P49411	Elongation factor Tu, mitochondrial	EFTU_HUMAN
u	91-219	87-219	Q96EH3	Mitochondrial assembly of ribosomal large subunit protein 1 (Homo sapiens)	MASU1_HUMAN
v	2-70, PNS	2-70, PNS	L0R8F8	MIEF1 upstream open reading frame protein (Homo sapiens)	MIDUO_HUMAN
w	70-156	70-156	O14561	Acyl carrier protein, mitochondrial (Homo sapiens)	ACPM_HUMAN
t3	62-91	62-91	P52815	39S ribosomal protein L12, mitochondrial	RM12_HUMAN
t4	62-90	62-90	P52815	39S ribosomal protein L12, mitochondrial	RM12_HUMAN
t5	62-91	62-91	P52815	39S ribosomal protein L12, mitochondrial	RM12_HUMAN
t6	63-89	63-89	P52815	39S ribosomal protein L12, mitochondrial	RM12_HUMAN
t7	Not present	129-198	P52815	39S ribosomal protein L12, mitochondrial	RM12_HUMAN

GTP, guanosine triphosphate; GDP, guanosine diphosphate; SAH, S -adenosylhomocysteine; SAM, S-adenosyl methionine; PNS 4'-phosphopantetheine

References

1. Rosenthal, P. B. & Henderson, R. Optimal Determination of Particle Orientation, Absolute Hand, and Contrast Loss in Single-particle Electron Cryomicroscopy. *J. Mol. Biol.* **333**, 721–745 (2003).
2. Baker, N. A., Sept, D., Joseph, S., Holst, M. J. & McCammon, J. A. Electrostatics of nanosystems: Application to microtubules and the ribosome. *Proc. Natl. Acad. Sci.* **98**, 10037–10041 (2001).
3. Seffouh, A. *et al.* Structural consequences of the interaction of RbgA with a 50S ribosomal subunit assembly intermediate. *Nucleic Acids Res.* **47**, 10414–10425 (2019).
4. Jaskolowski, M. *et al.* Structural Insights into the Mechanism of Mitoribosomal Large Subunit Biogenesis. *Mol. Cell* **79**, 629-644.e4 (2020).
5. Greber, B. J. *et al.* The complete structure of the 55S mammalian mitochondrial ribosome. *Science (80-.).* **348**, 303–308 (2015).
6. Desai, N. *et al.* Elongational stalling activates mitoribosome-associated quality control. *Science (80-.).* **370**, 1105–1110 (2020).
