# Supplementary material for: Structural basis for late maturation steps of the human mitoribosomal large subunit
Source: Nat Commun. 2021 Jun 16;12:3673. doi: 10.1038/s41467-021-23617-8 (PMC8209036; doi:10.1038/s41467-021-23617-8)
Supplement: Supplementary file 3 — Reporting Summary [file 41467_2021_23617_MOESM3_ESM.pdf]

nature research

Corresponding author(s): Joanna Rorbach, Martin Hällberg

Last updated by author(s): Apr 26, 2021

Reporting Summary

Nature Research wishes to improve the reproducibility of the work that we publish. This form provides structure for consistency and transparency in reporting. For further information on Nature Research policies, see Authors & Referees and the Editorial Policy Checklist.

Statistics

For all statistical analyses, confirm that the following items are present in the figure legend, table legend, main text, or Methods section.

n/a	Confirmed	
☒	☐	The exact sample size (*n*) for each experimental group/condition, given as a discrete number and unit of measurement
☒	☐	A statement on whether measurements were taken from distinct samples or whether the same sample was measured repeatedly
☒	☐	The statistical test(s) used AND whether they are one- or two-sided<br>*Only common tests should be described solely by name; describe more complex techniques in the Methods section.*
☒	☐	A description of all covariates tested
☒	☐	A description of any assumptions or corrections, such as tests of normality and adjustment for multiple comparisons
☒	☐	A full description of the statistical parameters including central tendency (e.g. means) or other basic estimates (e.g. regression coefficient) AND variation (e.g. standard deviation) or associated estimates of uncertainty (e.g. confidence intervals)
☒	☐	For null hypothesis testing, the test statistic (e.g. *F*, *t*, *r*) with confidence intervals, effect sizes, degrees of freedom and *P* value noted<br>*Give P values as exact values whenever suitable.*
☒	☐	For Bayesian analysis, information on the choice of priors and Markov chain Monte Carlo settings
☒	☐	For hierarchical and complex designs, identification of the appropriate level for tests and full reporting of outcomes
☒	☐	Estimates of effect sizes (e.g. Cohen's *d*, Pearson's *r*), indicating how they were calculated

Our web collection on statistics for biologists contains articles on many of the points above.

Software and code

Policy information about availability of computer code

Data collection	Cryo-EM data were collected from vitrified grids using a Krios G3i electron microscope (Thermofisher) operated at 300 kV and equipped with a K3 direct electron detector (Ametek). Automated data collection software was used during collection (EPU 2, Thermofisher) which was performed at 165,000× EFTEM SA magnification, yielding a calibrated pixel size of 0.51 Å. 1 sec exposures yielded a total fluency of 49 e-/Å2 in 60 frames, with targeted defocus values -0.3 to -1.1 µm.
Data analysis	Motion correction, CTF-estimation, Fourier cropping (to 1.02 Å/px), picking and extraction in 600 pixel boxes were performed on the fly using WARP 1.0.9. Cryosparc 2.15 and RELION 3.1 were used to run 2D classification and further analyses. The identification of unknown protein components in the density maps was done using the density based fold recognition pipeline available at https://www2.mrc-lmb.cam.ac.uk/groups/murshudov/content/em_fitting/em_fitting.html, based on Molrep 11.7.03, or using the DALI server available at http://ekhidna2.biocenter.helsinki.fi/dali/. The models of novel structures bound to the assembly intermediates were manually built with Coot 0.9.3. The starting models were generated with the SwissModel server available at https://swissmodel.expasy.org/ whenever coordinates were not available in the PDB. Stereochemical reciprocal space restrained refinement was performed using Refmac5 5.8.0267. The final model was validated using MolProbity in the Phenix suite (1.9.2-4158).

For manuscripts utilizing custom algorithms or software that are central to the research but not yet described in published literature, software must be made available to editors/reviewers. We strongly encourage code deposition in a community repository (e.g. GitHub). See the Nature Research guidelines for submitting code & software for further information.

Data

Policy information about availability of data

All manuscripts must include a data availability statement. This statement should provide the following information, where applicable:

- Accession codes, unique identifiers, or web links for publicly available datasets
- A list of figures that have associated raw data
- A description of any restrictions on data availability

Data availability section has been added to the manuscript: "The coordinates and corresponding cryo-EM maps were deposited in the Protein Data Bank (PDB) and

in the Electron Microscopy Data Bank (EMDB), respectively. For the GTPBP5KO mt-LSU assembly intermediate, the accession codes are 7O9M (atomic coordinates), EMD-12764 (main refinement map), EMD-12769 (particles with full MTG1 occupancy) and EMD-12770 (particles without MTG1). For the GTPBP5IP mt-LSU assembly intermediate, the accession codes are 7O9K (atomic coordinates), EMD-12763 (main refinement map), EMD-12767 (particles with full MTG1 occupancy) and EMD-12768 (particles with full mtEF-Tu occupancy). All other data can be obtained from the corresponding authors upon reasonable request."

Field-specific reporting

Please select the one below that is the best fit for your research. If you are not sure, read the appropriate sections before making your selection.

☒ Life sciences ☐ Behavioural & social sciences ☐ Ecological, evolutionary & environmental sciences

For a reference copy of the document with all sections, see nature.com/documents/nr-reporting-summary-flat.pdf

Life sciences study design

All studies must disclose on these points even when the disclosure is negative.

Sample size	37307 and 112076 micrographs were analysed for GTPBP5 KO and GTPBP5 IP mt-LSU structures, respectively. No statistical analysis has been performed.
Data exclusions	None. As indicated in the manuscript, about 70% of the particles from both datasets were representative of bad quality particles that were not further analysed.
Replication	All biochemical experiments (IP and gradient purification of mitoribosomes) that led to cryoEM sample preparation were performed at least 3 times. All attempts of replication were successful.
Randomization	Randomization is not relevant to this study as no statistical analysis were needed for this study.
Blinding	Blinding is not relevant to this study as structural data collection is not biased by the investigators.

Reporting for specific materials, systems and methods

We require information from authors about some types of materials, experimental systems and methods used in many studies. Here, indicate whether each material, system or method listed is relevant to your study. If you are not sure if a list item applies to your research, read the appropriate section before selecting a response.

Materials & experimental systems

n/a	Involved in the study	
☒	☐	Antibodies
☐	☒	Eukaryotic cell lines
☒	☐	Palaeontology
☒	☐	Animals and other organisms
☒	☐	Human research participants
☒	☐	Clinical data

Methods

n/a	Involved in the study	
☒	☐	ChIP-seq
☒	☐	Flow cytometry
☒	☐	MRI-based neuroimaging

Eukaryotic cell lines

Policy information about cell lines

Cell line source(s)	Flp-In Trex 293 cell line was purchased from Thermofisher Scientific.
Authentication	Flp-In™ T-REx™ 293 Cell Line (Catalog number: R78007) was purchased from Thermofisher Scientific. No authentication is required as these cells are Zeocin and Blasticidin resistant, in contrast to any other cell line.
Mycoplasma contamination	Cell line tested negative for mycoplasma contamination.
Commonly misidentified lines (See ICLAC register)	no commonly misidentified lines were used in this study.
